# Risk of sarcopenia and mobility of older adults during the COVID-19 pandemic: the longitudinal data from the REMOBILIZE study

**DOI:** 10.1007/s40520-024-02720-y

**Published:** 2024-03-28

**Authors:** Patricia Parreira Batista, Monica Rodrigues Perracini, Maria do Carmo Correia de Lima, Juleimar Soares Coelho de Amorim, Daniele Sirineu Pereira, Leani Souza Máximo Pereira

**Affiliations:** 1 Postgraduate Program in Rehabilitation Sciences, Universidade Federal de Minas Gerais (UFMG), Av. Pres. Antônio Carlos, 6.627. Pampulha, Belo Horizonte, MG Brazil; 2 Master’s and Doctoral Programs in Physical Therapy, Universidade Cidade de São Paulo (UNICID), São Paulo, SP Brazil; 3 Master’s and Doctoral Programs in Gerontology, Faculty of Medical Sciences, Universidade Estadual de Campinas (UNICAMP), Campinas, SP Brazil; 4 Physical Therapy Course, Instituto Federal do Rio de Janeiro, Rio de Janeiro, RJ Brazil; 5 Postgraduate Program in Health Sciences, Faculdade Ciências Médicas de Minas Gerais (FCMMG), Belo Horizonte, MG Brazil

**Keywords:** Aging, Risk of sarcopenia, Mobility limitation, Pandemic

## Abstract

**Background:**

We assessed whether clinical, functional and behavioral factors were associated with the decrease in mobility trajectories reported in older people at risk of sarcopenia (RS) and without risk of sarcopenia (NRS) during COVID-19 pandemic.

**Methods:**

We prospectively analyzed mobility trajectories reported in older adults with RS and NRS over 16-month follow-up (Remobilize study). The self-perceived risk of sarcopenia and mobility were assessed using the SARC-F and the Life-Space Assessment (LSA) tools, respectively. Gender, age, comorbidities, pain, functional limitation, physical activity (time spent in walking; min/week), and sitting time (ST; hours/day) were assessed. We used a multilevel model to determine changes in mobility between groups and over time.

**Results:**

Mobility was lower in RS than in NRS. Older people at RS, who were women, aged 70–79 years and 80 years or older, inactive, and with moderate to severe functional limitation experienced reduced mobility trajectories reported over the pandemic. For older people at NRS, trajectories with reduce mobility reported were experienced by women with comorbidities, for those with insufficient walking time and aged 70–79 years; aged 70–79 years and with ST between 5 and 7 hours/day; for those with insufficient walking time and increased ST; and for those with pain and increased ST.

**Conclusion:**

Mobility trajectories reported in older people at risk of sarcopenia were negatively influenced by insufficient level of physical inactivity and pre-existing moderate to severe functional limitation. Health and social interventions should be target to avoid mobility limitation during and after the COVID-19 pandemic.

**Supplementary Information:**

The online version contains supplementary material available at 10.1007/s40520-024-02720-y.

## Introduction

The COVID-19 pandemic has made social distancing a necessity, especially for older adults, who are at a higher risk of experiencing health complications, particularly when a vaccine is not yet available [1]. Social distancing increased the time indoors and sitting time (ST), discontinued health interventions, and reduced physical activity [2, 3]. Saunders et al. and Beauchamp et al. have evidenced that around 25% of community-dwelling older adults experienced a decline in physical activity levels during the pandemic [4, 5]. A study involving 387 older Americans during the initial three months of the pandemic also reported a decline in walking activity and an increase in sedentary time. The authors observed a positive and statistically significant association between ST and overweight as well as obesity [6]. During the early stages of the pandemic, the study observed a heightened rate of discontinuation of health-related interventions (including medical consultations, rehabilitation, and other modalities) [7]. This discontinuation pattern posed a significant risk of exacerbating chronic diseases, which are commonly prevalent among the older population [7]. As a result, home confinement exacerbates a detrimental cycle concerning body composition, muscle function, and mobility in older adults [2].

Mobility is the ability to move around in different environments (i.e., from home spaces and neighborhoods to regions outside the city) and it is also expressed as life-space mobility [8]. Mobility is a marker for healthy aging due to its association with functionality and social participation [8, 9]. Moreover, physical and psychosocial aspects, gender, culture, and lifestyle-related factors can affect life-space mobility [10]. Mobility limitation was associated with falls, cognitive decline, hospitalization, and higher mortality [8–12] and favored cardiovascular deconditioning and sarcopenia during the pandemic [2].

The research Network of Studies on Mobility in Aging (REMOBILIZE study) investigated the COVID-19 pandemic impacts over time on the health of older adults, especially mobility. Prior analyzes (n = 1,482) have demonstrated a significant decrease in mobility reported subsequent to the onset of the pandemic [13]. This result was associated with sex (men), age group (70–79 years), ethnicity (black), living alone, years of education (≥ four years), and higher income [13]. Studies demonstrate a significant association between reduced mobility and worsening quality of life in community-dwelling older adults [14, 15]. Conversely, increased mobility demonstrated significant associations with various physical and contextual determinants, as indicated by Saunders et al. [4]. Specifically, male gender, age, higher walking volume, absence of previous falls, neighborhood accessibility, reduced musculoskeletal pain, and improved health perception emerged as influential factors during the pandemic period. [4]. These findings further underscore the multidimensional nature of mobility.

These consequences intensified the aging process, reduced muscle mass and strength, and increased the systemic pro-inflammatory profile, anabolic resistance, catabolism, and risk of sarcopenia (RS) in older adults [2, 10]. Sarcopenia is a progressive multifactorial disorder characterized by reduced muscle mass and strength [16]. The European Working Group on Sarcopenia in Older People (EWGSOP2) proposes the use of the SARC-F questionnaire to assess the risk of sarcopenia (RS) in the initial stage of population screening, especially in primary care [16]. SARC-F is simple, self-reported, and endorsed by several scientific organizations and allows the early detection of risk of sarcopenia for referral to a detailed assessment of muscle mass and strength [16–19]. A positive SARC-F predicts functional decline, hospitalizations, progression to severe COVID-19, and death [20–23]. Moreover, older adults with pre-existing sarcopenia or at RS showed a worse clinical-functional prognosis during social distancing [2].

A cross-sectional analysis of the REMOBILIZE study, utilizing baseline data, reveals a prevalence of 17.1% for risk of sarcopenia during the pandemic (SARC-F). Older adults with RS showed significant decrease in mobility reported compared those without RS (NRS) [24]. Furthermore, after adjusting for sociodemographic factors, the risk of sarcopenia was associated with comorbidities, pain, moderate or severe functional limitation, walking time, ST, and mobility [24]. Given the challenging circumstances presented by the COVID-19 pandemic and the accompanying measures of social distancing, it becomes imperative to comprehend the impact of sarcopenia risk on the mobility patterns of older adults. Thus, this study aimed to assess mobility trajectories reported in community-dwelling older adults with RS and NRS during the pandemic and verify associated clinical, functional, and behavioral factors.

## Methods

### Study design and sample

This longitudinal study was part of the REMOBILIZE study that followed up on four COVID-19 pandemic waves [13]. Data were collected in four periods: T1 (baseline from May to July 2020), T2 (from August to October 2020), T3 (from February to April 2021), and T4 (from September to December 2021). Snowball sampling was adopted using an online survey (SurveyMonkey®), which was disclosed via social media and Whatsapp® groups in all Brazilian regions. Community-dwelling older adults (≥ 60 years), regardless of sex, ethnicity, or social class, were included. Those who were bedridden or institutionalized were excluded. Subjects with physical or mental disabilities were allowed to have a family member or caregiver answer their questions. [13]. This study was approved by the research ethics committee of the University of the City of São Paulo (no. 31592220.6.0000.0064), and the eligibility criteria and sample flowchart were previously published [13].

### Measures and instruments

Researchers prepared a questionnaire to collect self-reported sociodemographic and clinical information to characterize the sample. The study used the Functional Comorbidity Index (FCI) questionnaire to identify reported comorbidities, defined as the co-occurrence of two or more chronic diseases. [13, 25]. Pain was recorded during the assessments [13].

The SARC-F assessed the self-perceived risk of sarcopenia [16, 26] through four items related to muscle function: strength, assistance in walking, rising from a chair, and climbing stairs, along with one item addressing falls within the past year [16]. The total score ranged from 0 to 10, with a cut-off point ≥ 4 indicating the RS [16–19, 24]. This questionnaire was translated and validated for Brazilian older adults [24, 28]. A proxy-reported version was also validated [27]. Older adults were classified into the RS and NRS groups.

The Life-Space Assessment (LSA) evaluated self-reported mobility. The assessment considered five levels of complexity: mobility in the bedroom or sleeping area (level 1); in the external area of the residence (level 2); in the neighborhood (level 3); inside the city (level 4); and outside the city (level 5) [29]. Frequency per week and need for support (personal assistance or assistance of equipment) were registered. Each level generated a score, and the total score (0–120) was the sum of the five scores [29]. High scores indicated high mobility. LSA total score was used for description and analysis. The mobility trajectory, as assessed by the Life-Space Assessment (LSA) questionnaire, delineates the progression of reported life-space mobility over time.

The Brazilian OARS Multidimensional Functional Assessment Questionnaire (BOMFAQ) assessed functional limitations [30, 31]. This self-reported questionnaire comprised 15 activities of daily living (eight basic and seven instrumental activities). The items that reported “difficulty” or “need help” scored 1 point, and the total score ranged from 0 to 15. Older adults were classified into two categories: no or mild functional limitation (0 to 3 points) and moderate or severe functional limitation (≥ 4 points) [32].

The Incidental and Planned Activity Questionnaire (IPEQ), validated for older adults, assessed walking time to represent physical activity [33]. The questions referred to walking activities (planned or incidental). The score for each question was the product of the frequency per week and the time per occasion (hours/week), and their sum resulted in the total walking score, which was transformed to min/week. Older adults were classified as active (total score > 150 min/week), insufficiently active (score between 1 and 150 min/week), or inactive (those who did not perform any activity) [24, 34, 35].

One question evaluated self-reported sedentary time (ST), taking into account the time spent (hours/day) engaging in activities while sitting at home, including both weekdays and weekends. The possible answers were 4 h/day or less, 5 to 7 h/day, 8 to 10 h/day, and 10 h/day or more [24].

### Statistical analysis

The risk of sarcopenia and respective 95% confidence intervals (95%CI) at the four periods (T1 to T4) was computed. Data were described as mean and standard deviation or median and interquartile ranges for continuous data, according to data normality, and absolute or relative frequency for categorical variables. Descriptive data of sample loss were recorded.

The longitudinal multilevel model quantified the variance of mobility (LSA total score) among older adults and periods within groups and the differences between older adults with associated factors. The model comprised a structural (deterministic) and a stochastic (random) component. The effect of independent covariates (age group, sex, functional limitation, comorbidities, physical activity, ST, and pain) was calculated using the intercept and time slope (T1 to T4). The step-wise procedure (Wald test) tested the covariates for inclusion in the model, assuming a 10% significance.

Analyses were performed in the Linear and Nonlinear Mixed Effects Models (NLME) package of Software R, estimator = maximum likelihood (ML) (supplemental material). In model 1 (random intercept), the estimated variance among older adults and intraclass correlation coefficient (ICC) (ρ) were calculated. In model 2 (non-conditional growth model), time (*Tij*) was added to model 1, allowing calculation of the variability among older adults over time (mobility over time in each group). Model 3 included a polynomial effect (degree 3) to model the relationship between mobility and time (quadratic and cubic term, 10% significance), analyzing the estimated coefficients and the intercept of the mean trajectory in LSA in each group. Model 4 added the independent covariates and their interaction with time. All assumptions on residuals of the longitudinal multilevel model were checked for each group (RS and NRS) (homoscedasticity and normality of residuals; Supplementary information 1).

Assuming that ${Y}_{ij}$ is the observed response (mobility) of an older adult $i$ at time $j$, and ${X}_{1}$, ${X}_{2}$,…,${X}_{p}$ are $p$ covariates, the general equation of the model was given by:

$${Y}_{ij}={\Pi }_{0i}+{\Pi }_{1i}{T}_{ij}{+\Pi }_{2}{T}_{ij}^{2}{+\Pi }_{3}{T}_{ij}^{3}+{\varepsilon }_{ij} (\mathrm{level }1\mathrm{ model})$$

with the following submodels at level 2: $$\left\{\begin{array}{c}{\Pi }_{0i}={\gamma }_{00}+{\gamma }_{01}{X}_{1}+\dots + {\gamma }_{0p}{X}_{p}+ {\xi }_{0i}\\ {\Pi }_{1i}={\gamma }_{10}+{\gamma }_{11}{X}_{1}+\dots + {\gamma }_{1p}{X}_{p}+ {\xi }_{1i}\end{array}\right.$$

For level 1:

- ${\Pi }_{0i}$ is the random intercept (represents the true mobility at T1 for $i$);
- ${\Pi }_{1i}$ is the random slope (represents the true change in $i$ trajectory during the period);
- ${\Pi }_{2}$ and ${\Pi }_{3}$ are the quadratic and cubic terms that allow modeling a polynomial effect to represent mobility in a nonlinear model;
- ${\varepsilon }_{ij}$ represents the portion of $i$ that was unable to predict at $j$.

At submodules in level 2:

- ${\gamma }_{00}$ and ${\gamma }_{10}$, level 2 intercepts represented the population mean of mobility at T1 and its change, respectively, when all $p$ covariates were equal to zero.
- ${\gamma }_{0k}(k=\mathrm{1,2},\dots ,p)$ represents the effect of *k-th* covariate on the slope, providing increments (or decrements) to mobility at T1.
- ${\xi }_{0i}$ and ${\xi }_{1i}$, level 2 residuals represent the portions of mobility at T1 and changes in mobility that were not explained at this level. They represent deviations in the changes of older adults around their respective mean trends.

In this representation, the level 1 model described how each older adult changed over time, and the level 2 model related to differences among older adults with associated factors. Model 4 for the group's RS and NRS is described in Supplementary information 1.

## Results

The study included 1,482 older adults; 254 were in RS group at T1. Sample loss comprised 642 older adults, from which 122 were RS: 78.7% were women, 40.2% were aged 80 years or older, 85.2% presented comorbidities, 75.4% presented moderate or severe functional limitation, and 73.8% were inactive. Considering the NRS group sample loss, 70.8% were women, 59.2% were aged 60 to 69 years, 53.3% presented comorbidities, 88.8% presented no or mild functional limitation and 43.8% were inactive. Supplementary information 2 presents the sample flowchart.

**Fig. 1:**
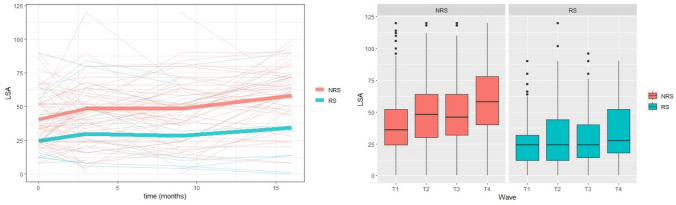
Analysis of reported mean mobility trajectories in living spaces in older adults with and without risk of sarcopenia. Where **a** corresponds to the visualization of the trajectories of 100 individuals in the sample by random selection and smoothing of means over time (n = 1482; LSA); and **b** LSA boxplot by wave and by group, with and without risk of sarcopenia

The prevalence of RS was 17.1% (95%CI 15.25 to 19.15), 17.1% (95%CI 15.04 to 19.37), 16.5% (95%CI 14.27 to 18.84), and 15.7% (95%CI 13.3 to 18.35) for T1, T2, T3, and T4, respectively. From RS group, 83.1% were women, 42.5% were 80 years or older, 39% were married, 31.5% had one to four years of education, and 39.8% had nine or more years of education. From NRS group, 9.9% were 80 years or older, 72.1% were women, 56.7% were married, 16.5% had one to four years of education, and 65.2% had nine or more years of education. Table 1 shows the descriptive data and mobility reported over time. Figure 1 represents the mean mobility trajectories and the boxplot (T1–T4).

**Table 1:** Descriptive data of the groups without and with risk of sarcopenia

		NRS group				RS group			
		T1	T2	T3	T4	T1	T2	T3	T4
Age, % (years)	60–69	61,3				31,5			
	70–79	28,8				26,0			
	80 years and older	9,9				42,5			
Sex, %	Men	27,9				16,9			
	Women	72,1				83,1			
Comorbidities (≥ 2), %		50,4				87,4			
BOMFAQ^*b*^ (4 pts +), %		10,3	11,1	11,4	10,8	73,6	75,7	67,4	65,6
Walking (total), %	Inactive	46,7	33,6	31,6	27,4	74,5	64,3	61,2	60,3
	Insufficiently active	33,6	37,0	39,2	38,4	21,4	24,9	32,0	34,1
	Active	19,7	29,4	29,2	34,2	4,1	10,8	6,8	5,6
Sitting time, % (hours/day)	< 4	48,6	49,0	46,8	52,1	28,8	31,9	24,5	35,7
	5–7	31,0	30,9	31,9	29,3	31,1	27,0	29,3	25,4
	8–10	12,6	10,8	12,1	11,6	16,9	17,3	22,5	22,2
	> 10	7,8	9,3	9,2	7,0	23,2	23,8	23,8	16,7
Pain (yes), %		21,6	23,5	23,2	21,4	55,5	49,7	53,1	46,1
LSA, score total med (IQR)		36 (24–52)	48 (30–64)	46 (32–64)	58 (40–78)	24 (12–32)	24 (12–44)	24 (14–40)	27,5 (17.8–52)

T1 BASELINE; T2 3-month follow-up; T3 9-month follow-up; T4 16-month follow-up; Med median; IQR interquartile range 1st and 3rd; BOMFAQ Brazilian OARS Multidimensional Functional Assessment Questionnaire; (score of four points or more refers to the presence of moderate to severe functional limitation); walking the sum of walking as exercise and utility in the previous week (min/week); Sitting Time at home; LSA Life-Space Assessment

**Fig. 2:**
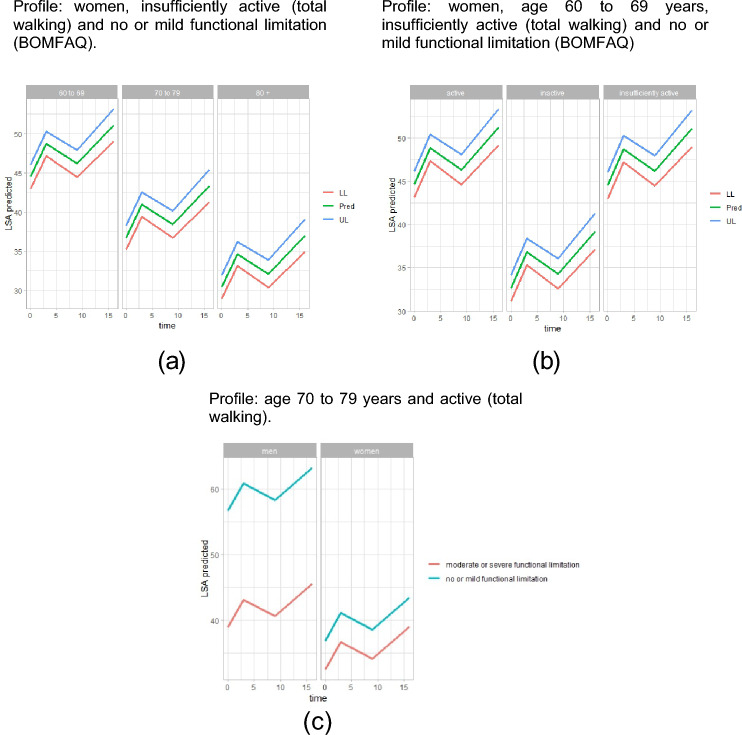
Analysis of predicted trajectories of mobility (LSA) of older adults at risk of sarcopenia (Model 4). Profile: women, insufficiently active (total walking) and no or mild functional limitation (BOMFAQ). Profile: women, age 60–69 years, insufficiently active (total walking) and no or mild functional limitation (BOMFAQ). Profile: age 70–79 years and active (total walking). Fixing the profile of older adults at risk of sarcopenia; mobility trajectories by **a** age group; **b** total walk; and **c** the effect of the interaction between sex and the presence of functional limitation (BOMFAQ). Where **a** and **b** illustrate the 95% confidence intervals (lower and upper limits); Pred Predicted Trajectory; LL Lower Limit; UL Upper limit; Fem Female

Table 2 presents the results of the longitudinal multilevel model for mobility in RS group. Model 3 was significant on the quadratic (${\Pi }_{2}$) and cubic (${\Pi }_{3}$) (*p* < 0.01), indicating a nonlinear pattern of mobility over time and components with a small variance in model 4 (${\widehat{\sigma }}_{\varepsilon }^{2}$ e ${\widehat{\sigma }}_{0}^{2}$). Age group, walking time, sex, and functional limitations were significantly correlated with changes in mobility in RS group. The predicted mobility reported for the RS is presented in Fig. 2. Older and inactive adults presented lower mean mobility trajectories than those aged 60–69 years and active (Figs. 2a and b). In contrast, women presenting moderate or severe functional limitation showed the mean mobility reduced by 24.19 units after fixing age and walking time (estimation using model 4; Fig. 2c). Other covariates did not influence changes in mobility over time in RS group.

**Table 2:** Results of the adjustment of longitudinal multilevel models for the trajectory of LSA in older adults at risk of sarcopenia (RS)

		Parameter	Model 1	Model 2	Model 3	Model 4
Fixed effects						
Initial LSA, p_0i_	Intercept	γ_00_	30,71 (1,91)***	27,70 (1,899)***	26,36 (2,056)***	64,43 (6,25)***
	Sex (ref: men)					–
	Women	γ_01_				− 19,78 (5,74)***
	Age (ref: 60—69 years)					–
	(70–79 years)	γ_02_				− 7,77 (3,67)*
	(80–89 years)	γ_03_				− 14,08 (3,19)***
	BOMFAQ (ref: none or mild)					–
	(moderate to severe)	γ_04_				− 17,73 (5,40)**
	Total walk (ref: active)					–
	(inactive)	γ_05_				− 12,01 (3,28)***
	(insufficiently active)	γ_06_				− 0,148 (3,21)
	Sex*BOMFAQ (men* none or mild)					–
	(women * moderate to severe)	γ_07_				13,32 (5,84)*
LSA rate of change, p_1i_	Intercept	γ_10_		0,431 (0,122)***	3,020 (0,966)**	2,50 (0,966)*
Quadratic term, p_2_		π_2_			− 0,476 (0,161)**	− 0,419 (0,161)**
Cubic term, p_3_		π_3_			0,020 (0,007)**	0,018 (0,007)**
Variance components						
	Intra-individuals	σ_e_^2^	273,59	127,41	120,86	114,52
	Between individuals: in the initial LSA	σ_0_^2^	157,93	234,86	238,63	87,248
	Between individuals: at the rate of change	σ_1_^2^		0,424	0,468	0,494
Fit quality statistics						
AIC			2901,7	2882	2877	2808
BIC			2913,2	2905	2908	2865
TRV (model a, Model a-1)			–	25,64***	9,07*	83,15***

These models predict the LSA between baseline and the fourth survey wave (0–16 months) as a function of time (level 1) and covariates (level 2). BOMFAQ Brazilian OARS Multidimensional Functional Assessment Questionnaire; score of four points or more refers to the presence of moderate to severe functional limitation; LSA Life-Space Assessment; AIC Akaike Information Criterion. BIC Bayesian Information Criterion. LRT Likelihood Ratio Test

Obs.: Run in NLME package of Software R, estimator = ML

~ *p* < 0,10; * *p* < 0,05; ** *p* < 0,01; *** *p* < 0,001

Table 3 displays the results of a multilevel analysis for reported mobility trajectories within the NRS group, including the significant polynomial effect (*p* < 0.001) and reduced variance components in model 4 (${\widehat{\sigma }}_{\varepsilon }^{2}$ and ${\widehat{\sigma }}_{0}^{2}$). All variables showed significance (whether isolated or interacted) and were included in model 4.

**Table 3:** Results of the adjustment of longitudinal multilevel models for the mean trajectory LSA in older adults without risk of sarcopenia (NRS)

		Parameter	Model 1	Model 2	Model 3	Model 4
Fixed effects		γ				
Initial LSA, p_0i_	Intercept	γ_00_	48,99 (0,852)***	42,45 (0,932)***	39,83 (1,044)***	59,3 (2,432)***
	Sex (ref: men)					–
	women	γ_01_				− 7,30 (2,151)***
	Age (ref: 60—69 years)					–
	(70–79 years)	γ_02_				− 4,54 (2,638) ~
	(80–89 years)	γ_03_				− 13,48 (4,493)**
	BOMFAQ (ref: none or mild)					–
	(moderate to severe)	γ_04_				− 2,36 (2,464)
	Comorbidities (ref: No)					–
	(yes)	γ_05_				3,10 (2,84)
	Total Walk (ref: active)					–
	(inactive)	γ_06_				− 10,02 (2,242)***
	(insufficiently active)	γ_07_				− 2,93 (2,141)
	Sitting Time (ref: ≤ 5 h/day)					–
	(5—7 h/day)	γ_08_				− 2,40 (2,321)
	(8—10 h/day)	γ_09_				0,74 (3,867)
	(> = 10 h/day)	γ_010_				1,43 (4,513)
	Pain (ref: no)					–
	(yes)	γ_011_				− 1,91 (1,511)
	Sex*Comorbidities					–
	(women *yes)	γ_012_				− 6,59 (3,301)*
	Age*Total walk(60–69 years* active)					–
	(70–79 years* inactive)	γ_013_				− 3,85 (2,979)
	(80–89 years*inactive)	γ_014_				− 1,57 (4,629)
	(70–79 years* insufficiently active)	γ_015_				− 4,77 (2,744) ~
	(80–89 years* insufficiently active)	γ_016_				2,22 (4,516)
	Age*Sitting Time(60–69 years* < 4 h/day)					–
	(70–79 years*5—7 h/day)	γ_017_				5,38 (2,344)**
	(80–89 years*5—7 h/day)	γ_018_				− 0,27 (4,066)
	(70–79 years*8—10 h/day)	γ_019_				2,33 (3,696)
	(80–89 years*8—10 h/day)	γ_020_				5,23 (4,583)
	(70–79 years* > = 10 h/day)	γ_021_				− 1,62 (4,448)
	(80–89 years* > = 10 h/day)	γ_022_				0,54 (5,219)
	Total walk * Sitting Time (active* < 4 h/day)					–
	(inactive*5—7 h/day)	γ_023_				− 5,91 (2,507)*
	(insufficiently active *5—7 h/day)	γ_024_				− 6,98 (2,436)**
	(inactive*8—10 h/day)	γ_025_				− 14,81 (3,925)***
	(insufficiently activ *8 –10 h/day)	γ_026_				− 9,98 (4,002)*
	(inactive* ≥ 10 h/day)	γ_027_				− 8,82 (4,691) ~
	(insufficiently active* ≥ 10 h/day)	γ_028_				− 2,92 (4,608)
	Sitting Time *pain < 4 hs					
	(5–7 h*yes)	γ_029_				1,98 (2,318)
	(8–10 h*yes)	γ_030_				5,91 (3,098) ~
	(≥ 10 h*yes)	γ_031_				1,21 (3,615)
LSA rate of change, p_1i_	Intercept	γ_10_		0,935 (0,068)***	5,388 (0,584)***	4,02 (0,595)***
	BOMFAQ (ref: none or mild)					–
	(moderate to severe)	γ_11_				− 0,51 (0,236)*
	Total Walk (ref: active)					–
	(inactive)	γ_12_				0,34 (0,182) ~
	(insufficiently active)	γ_13_				0,25 (0,174)
	Sitting Time (ref: ≤ 5 h/day)					–
	(5–7 h/day)	γ_14_				0,31 (0,159) ~
	(8–10 h/day)	γ_15_				− 0,09 (0,232)
	(> = 10 h/day)	γ_16_				0,05 (0,274)
Quadratic term, p_2_		π_2_			− 0,784 (0,097)***	− 0,624 (0,095)***
Cubic term, p_3_		π_3_			0,032 (0,004)***	0,026 (0,004)***
Variance components						
	Intra-individuals	σ_e_^2^	336,84	270,74	253,79	230,91
	Between individuals: in the initial LSA	σ_0_^2^	269,57	267,09	276,87	177,64
	Between individuals: at the rate of change	σ_1_^2^		0,45	0,56	0,58
Fit quality statistics						
AIC			17,606	17,395	17,336	17,106
BIC			17,623	17,428	17,380	17,357
LRT (Model a, Model a-1)			–	217,09***	63,09***	303,93***

These models predict the LSA between baseline and the fourth survey wave (0–16 months) as a function of time (level 1) and covariates (level 2). BOMFAQ Brazilian OARS Multidimensional Functional Assessment Questionnaire; score of four points or more refers to the presence of moderate to severe functional limitation; LSA Life-Space Assessment; AIC Akaike Information Criterion. BIC Bayesian Information Criterion. LRT Likelihood Ratio Test

Obs.: Run in the NLME package of Software R, estimator = ML

~ *p* < 0,10; *p < 0,05; **p < 0,01; ***p < 0,001

In NRS group, a significant interaction was observed between age group and physical activity, age group and ST, sex and comorbidities, walking time and ST, and pain and ST. Women with comorbidities presented the mean mobility trajectories reduced by 10.79 units compared with men without comorbidities. Insufficiently active older adults and older adults with ST from 5 to 7 h/day presented mobility reduced by 12.31 units compared with active ones and those with ST of 4 h/day or less. In addition, inactive older people with ST from 8 to 10 h/day presented mobility reduced by 24.09 units considering the cited reference. Insufficiently active older adults aged 70–79 presented the mean mobility trajectories reduced by 12.24 units compared with active older adults (*p* < 0.10). Whereas, older people in this age group with ST from 5 to 7 h/day presented the mean mobility trajectory reduced by 1.56 units compared with those aged 60 to 69 years and with ST of 4 h/day or less (*p* < 0.01).

Regarding changes in reported mobility over time, only moderate or severe functional limitation reduced the mean mobility trajectories by 0.51 units per month relative to no or mild functional limitation in NRS (*p* < 0.05). Supplementary information 3 shows the predicted mobility for NRS group. Further details and the influence of covariates on changes in mobility are presented in Table 3.

## Discussion

The prevalence of RS was similar among study periods (17.1% at T1 and T2, 16.5% at T3, and 15.7% at T4), suggesting low sensitivity of SARC-F to the changes in muscle function and falls during the COVID-19 pandemic. Possible explanations are that social distancing reduced the exposure to risk behaviors for falls, and the study period (16 months) was insufficient to impair muscle function since older adults continued to perform their daily activities at home. Moreover, the sample presented a high level of education and income, possibly providing more access to health prevention and education using different means of communication during the pandemic.

The RS group reported decreased mobility during the COVID-19 pandemic. A pre-pandemic study with 391 community-dwelling older adults (69.8% women) observed an inverse and significant correlation between risk of sarcopenia (SARC-F) and mobility [36]. The present study observed substantial variability in reported mobility trajectories among older adults within and between the assessed periods (T1 to T4) (Fig. 1). Thus, the identification of subgroups within the RS and NRS groups, along with their associated independent factors, may provide insights into the underlying causes of changes in mobility during the pandemic.

Age group, walking time, sex, and functional limitations were significantly associated with changes in mobility in RS group. Older adults aged 70–79 years (7.77 units) and 80 years or older (14.08 units) presented lower mean mobility trajectories than those aged 60–69 years. These data confirm the negative and progressive effect of aging on mobility in RS. Pre-pandemic studies confirm the relationship between RS and older age [21, 22, 37]. Wu et al. (2016) reported a significant association between RS and older age at a 4-year follow-up in community-dwelling older adults in China [23]. In a multiethnic cohort, researchers identified RS in 3.8% of men and 7.6% of women younger than 70 years, as well as in 31.4% of men and 46.8% of women aged 90 years and older [37]. Longitudinal studies (6- and 9-year follow-ups) confirmed age as a risk factor for the progression of sarcopenia in community-dwelling older adults [37–39]. The deleterious repercussions on muscle mass and function related to advancing age contribute to the findings on the mean mobility trajectory reported in the RS group.

Walking time (physical activity) showed an isolated effect on mobility in RS group. Inactive older adults presented lower mean mobility trajectories (12.01 units) than active ones in RS, while insufficiently active older adults did not differ from active ones. Pre-pandemic studies demonstrated that no or insufficient physical activity was independently correlated with RS and confirmed sarcopenia in community-dwelling older adults [40–43]. Kwan et al. [44] reported that moderate to vigorous physical activity was a protective factor for sarcopenia in community-dwelling older adults aged 85 years or older in China during the pandemic. They also indicated that physical inactivity was associated with a high prevalence of RS during a one-year follow-up. However, mobility was not correlated with risk of sarcopenia, suggesting home-based or neighborhood physical activity [44]. This population has different habits regarding home-based physical activity [45], which are uncommon in Brazil. In our study, 74.5% of RS group were inactive and 21.4% insufficiently active at T1, and 60.3% were inactive and 34.1% insufficiently active at T4.

Moderate or severe functional limitation and sex showed significant interaction in the reported mean mobility trajectory over time in RS group. Men with moderate or severe functional limitation presented a lower mean mobility trajectory (17.73 units) than men with no or mild functional limitation, the latter reaching the highest predicted mobility. In comparison, women with moderate or severe functional limitation showed more impairment in mobility. Studies evidenced a higher RS for women than men [23, 37, 40–42] and an association with functional limitations [40]. Low mobility in community-dwelling older adults demonstrated a significant relationship with sex (female) and functional limitations. [46, 47]. Matsuda et al. (2022) demonstrated the distinct influence of sex on reported mobility, while also highlighting the direct impact of muscle mass index and social isolation on mobility in both males and females. [46]. Older adults with reduced mobility (LSA < 56 units) presented a two-fold odds ratio for reduced instrumental activities of daily living during a one-year follow-up [47]. Women, regardless of the presence of functional limitations, and men with moderate or severe functional limitations should undergo regular reassessment due to their heightened vulnerability to muscle catabolism and functional limitations over time. Psychological factors, coupled with adherence to social distancing, insufficient support, and fear of contamination, may influence to the onset or exacerbation of functional and mobility limitations.

A 10-unit change in the LSA total score was regarded as clinically relevant in community-dwelling older adults [15]. In this study, no group recovered mobility in the 16-month follow-up, which is consistent with previous findings. [13, 14]. Another REMOBILIZE study showed a reduction in LSA total score of over 20 units after the onset of the COVID-19 pandemic [13]. In the present study, RS group increased the LSA total score from 24 units at T1 to 27.5 units at T4. Thus, despite vaccination (January 2021) and decreased transmissibility rates, RS group slightly increased mobility compared with NRS (LSA = 36 units at T1; 58 units at T4) during the pandemic. Additionally, a cutoff point of 60 points in the LSA total score for the identification of sarcopenia risk has been suggested [36]. Interestingly, in the present study, even the NRS group did not attain this score at T4, indicating a greater propensity for RS and sarcopenia. RS highly predisposes sarcopenia progression and negative repercussions, justifying the scientific efforts to understand its relationship with mobility [2].

The longitudinal analysis in the NRS group revealed a dynamic adjusted model that incorporated interactions among covariates to explain the variability in reported mobility. Women with comorbidities presented a lower mean mobility trajectory than men without comorbidities. A recent study reported higher life-space mobility in men (n = 106; 77.5 ± 5.3 years) than in women (n = 188 women; 78.4 ± 5.5 years) [46]. The study reported that social distancing, muscle mass index (adjusted for height), lower limb muscle strength, and body mass index influenced mobility in men. While age, mobility (Timed Up and Go test), self-efficacy for falls, muscle mass index, and social distancing influenced life-space mobility in women [43]. Morros-González et al. (2021) demonstrated more women with low LSA total score (57.6%) than men (32.3%) in 22,995 older adults in Colombia, using a cut-off point of 60 units for low mobility [50]. Two or more chronic diseases presented a 1.32-fold (95%CI 1.06—1.63) odds ratio to reduce mobility, regardless of age group, sex, income, or access to health care. Hypertension, diabetes mellitus, osteoarthritis, and mental illness were significantly associated with low mobility [48]. In the present study, men in NRS group showed greater recovery and occupancy in living spaces over time during the pandemic than women, especially considering women with comorbidities.

Although sedentary behavior and physical activity are distinct lifestyles, they present similar repercussions on muscle function and risk of sarcopenia in older adults [41–44]. Tzeng et al. [43] reported an association between insufficient physical activity and ST (≥ 7 h/day) and risk of sarcopenia (SARC-F ≥ 4 points; OR: 5.14 and 1.98, respectively). During the pandemic, a decrease in the practice of physical activity was observed among community-dwelling older adults [4, 5]. In their study, Lefferts et al. [6] observed a decline in physical activity and an increase in sedentary behavior during the initial months of the pandemic, with a subsequent return to pre-pandemic levels after a 12-month follow-up period. In the present study, walking time and ST interacted in the adjusted model for NRS group. Inactive older adults with ST from 5 to 7 h/day (LSA = 18.33) and 8 to 10 h/day (LSA = 24.09) showed a reduction in the reported mean mobility trajectory of 18.33 and 24.09 units, respectively (*p* < 0.05). This finding suggests that engaging in regular walking, whether for utilitarian purposes or exercise, even at levels meeting the minimum recommendations, may help mitigate the detrimental effects of sedentary behavior on health and well-being.

NRS aged 70–79 years showed different behavior in reported mobility. Older adults with ST from 5 to 7 h/day reduced the mean mobility trajectory by 1.56 units compared with those with ST of 4 h/day or less (*p* < 0.01). Insufficiently active older adults aged 70–79 years showed a mean mobility trajectory 12.24 units lower than those active and aged 60–69 years (*p* < 0.10). In addition, mobility was not correlated with ST and walking time in older adults aged 80 years or more. This result may be due to greater home-based mobility, regardless of the pandemic. Findings from the reported mobility assessment during the pandemic underscore the impact of individuals' lifestyle behaviors and the interplay between physical activity and sedentary, which may serve as a potential predictor for increased risk of sarcopenia.

Moderate or severe functional limitation, physical inactivity, and ST (5–7 h/day) influenced reported mobility over time in NRS group. Older adults with moderate or severe functional limitations exhibited a monthly mean mobility trajectory reduction of 0.51 units compared to those with no or mild functional limitations during the analyzed period (*p* < 0.05). Even within the NRS group, it is imperative to incorporate preventive strategies during and post-pandemic to improve lifestyle and enhance muscle function. Data from The Longitudinal Aging Study Amsterdam (n = 1,119 older adults; 62–98 years) showed that half of older adults reported being less active during the pandemic, particularly those of younger age, women, and with functional limitations [14]. A reduction in physical activity by 1 h/week or more was associated with poor sleep quality, depressive symptoms, and anxiety [49]. Hence, long-term health interventions targeting older adults should take into account the lifestyle changes resulting from the pandemic.

The elevated prevalence of physical inactivity and the presence of functional limitations observed in individuals with RS, coupled with prolonged periods of sedentary behavior and restricted mobility at home, lead to an accelerated progression of sarcopenia. These lifestyle habits can initiate a vicious cycle of muscle catabolism, influenced by reduced muscle protein synthesis, greater mitochondrial dysfunction, anabolic resistance, insulin resistance, and oxidative and pro-inflammatory stress [2]. The intrinsic relationship between muscle and bone mass leads to greater susceptibility to bone fragility and osteoporosis. The co-existence of sarcopenia and osteoporosis, known as osteosarcopenia, amplifies common negative repercussions, including functional disability, falls, associated bone fractures, pain, and increased morbidity and mortality [50]. On the other hand, the World Health Organization proposes “The Decade of Healthy Aging 2021—2030,” which comprises a multidimensional and intersectoral collaborative plan to promote healthy aging [51]. Given the advent of the Covid-19 pandemic, the demand for action to strengthen and facilitate access to early detection of musculoskeletal decline, targeting physical rehabilitation and preventive measures must be reinforced [52].

This study is the first to investigate the trajectories of reported mobility in older adults with risk of sarcopenia (RS) and without risk of sarcopenia (NRS) during the pandemic (16-month follow-up). However, this study had limitations. The range of the online survey was possibly limited, reaching only older adults with better education, technological skills, family support, and care. While the Proxy-reported SARC-F is a valid instrument for the aging population [27], the potential for proxy-reporting the questionnaires, particularly for individuals with physical or mental disabilities, may have influenced our results. Nonetheless, the feasibility and safety in a pandemic scenario supported the methodology of this study. Thus, these results highlight the need to promote physical activity via social media, which should be encouraged to prevent sarcopenia in pandemic outbreaks.

## Conclusion

Throughout the 16-month duration of the pandemic, older adults exhibited distinct reported mobility trajectories, effectively distinguishing individuals at risk of sarcopenia from those without such risk. Women, moderate or severe functional limitation, older age, and physical inactivity influenced mobility trajectory in RS group during the pandemic. Identifying modifiable factors in mobility trajectories enables preventive actions, monitoring, and targeted physical interventions in older adults with worse predicted mobility, mitigating negative repercussions associated with the period of home confinement. In addition, our findings make it possible to manage groups at greater risk of mobility limitation, including individuals with and without risk of sarcopenia, during possible new pandemic outbreaks.

### Supplementary Information

Below is the link to the electronic supplementary material.

Supplementary file1 (DOCX 357 kb)
