# Supplementary material for: Risk of sarcopenia and mobility of older adults during the COVID-19 pandemic: the longitudinal data from the REMOBILIZE study
Source: Aging Clin Exp Res. 2024 Mar 28;36(1):80. doi: 10.1007/s40520-024-02720-y (PMC10978643; doi:10.1007/s40520-024-02720-y)
Supplement: Supplementary file 1 — Supplementary file1 (DOCX 357 kb) [file 40520_2024_2720_MOESM1_ESM.docx]

**Supplementary Information 3:**

**Figure 2. Analysis of predicted trajectories of mobility (LSA) of older adults without risk of sarcopenia (Model 4).**

Profile: women, moderate to severe limitation (BOMFAQ), 10 hours or more/day of sitting time at home, absence of comorbidities and presence of pain.

Profile: Age 70-79 years, insufficiently active (total walking), moderate to severe limitation (BOMFAQ), 5-7 hours/day sitting at home and presence of pain.


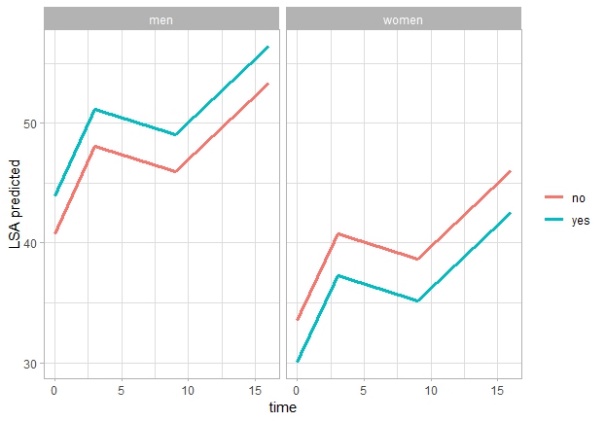

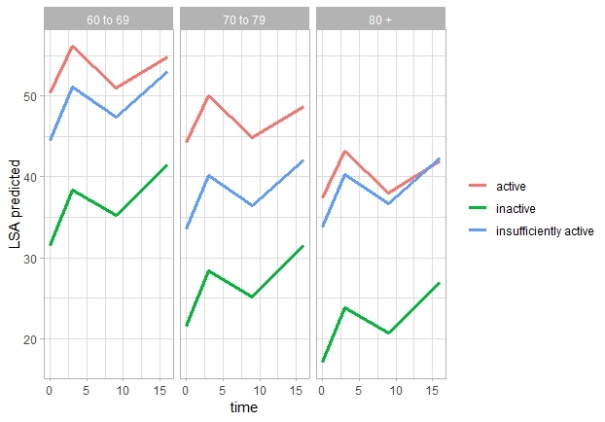


(a) (b)

Profile: Inactive (total walking), moderate to severe limitation (BOMFAQ), 5-7 hours/day sitting time at home and no pain.

Profile: women; age 70-79 years, moderate to severe limitation (BOMFAQ), presence of comorbidities and pain.


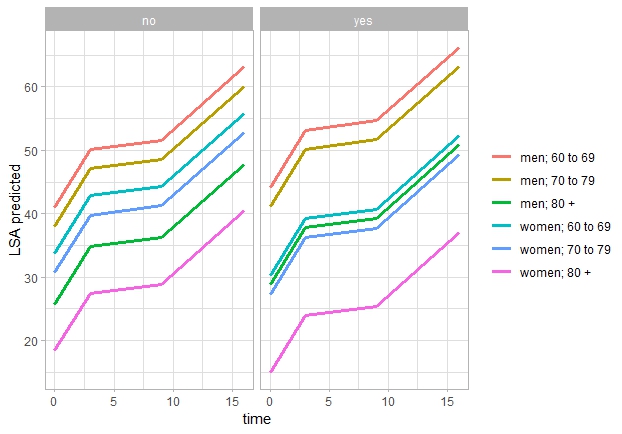

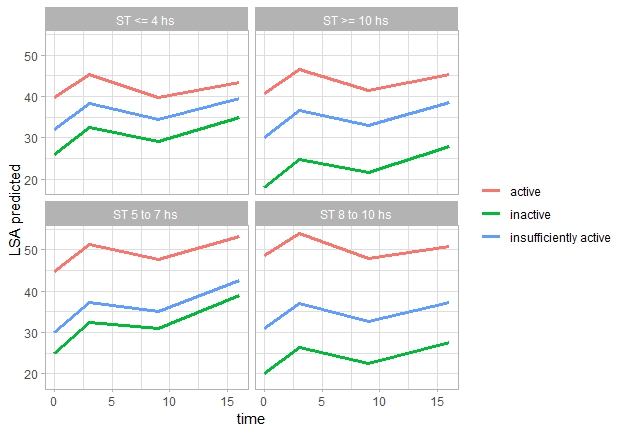


(c) (d)

Fixing the profile of older adults without risk of sarcopenia, we have a predicted trajectory of LSA on: a. the effect of the interaction of the variables sex and presence of comorbidities; b. the interaction effect of age and total walk; c. interaction effect of total walking and sitting time at home; d. effect of the interaction of sex, age and presence of comorbidities. LSA Life-Space Assessment.
